# Structure of the N-Terminal Mlp1-Binding Domain of the *Saccharomyces cerevisiae* mRNA-Binding Protein, Nab2

**DOI:** 10.1016/j.jmb.2007.11.087

**Published:** 2008-02-29

**Authors:** Richard P. Grant, Neil J. Marshall, Ji-Chun Yang, Milo B. Fasken, Seth M. Kelly, Michelle T. Harreman, David Neuhaus, Anita H. Corbett, Murray Stewart

**Affiliations:** 1 MRC Laboratory of Molecular Biology, Hills Road, Cambridge CB2 0QH, UK; 2 Department of Biochemistry, Emory University School of Medicine, Rollins Research Center, Atlanta, GA, USA

**Keywords:** Nab2, nuclear abundant poly(A) RNA-binding protein 2, Mlp1, myosin-like protein 1, NPC, nuclear pore complex, hnRNP, heterogeneous nuclear ribonucleoprotein, CT-Mlp1, C-terminal globular domain of Mlp1, GST, glutathione *S*-transferase, rmsd, root-mean-square deviation, NOE, nuclear Overhauser enhancement, TLS, translation/libration/screw, DBD, DNA-binding domain, AD, activation domain, PBS, phosphate-buffered saline, EDTA, ethylenediaminetetraacetic acid, HSQC, heteronuclear single quantum coherence, NOESY, nuclear Overhauser enhancement spectroscopy, PDB, Protein Data Bank, mRNA, Nab2, PWI, nuclear export, Mlp1

## Abstract

*N*uclear abundant poly(*A*) RNA-*b*inding protein 2 (Nab2) is an essential yeast heterogeneous nuclear ribonucleoprotein that modulates both mRNA nuclear export and poly(A) tail length. The N-terminal domain of Nab2 (residues 1–97) mediates interactions with both the C-terminal globular domain of the nuclear pore-associated protein, myosin-like protein 1 (Mlp1), and the mRNA export factor, Gfd1. The solution and crystal structures of the Nab2 N-terminal domain show a primarily helical fold that is analogous to the PWI fold found in several other RNA-binding proteins. In contrast to other PWI-containing proteins, we find no evidence that the Nab2 N-terminal domain binds to nucleic acids. Instead, this domain appears to mediate protein:protein interactions that facilitate the nuclear export of mRNA. The Nab2 N-terminal domain has a distinctive hydrophobic patch centered on Phe73, consistent with this region of the surface being a protein:protein interaction site. Engineered mutations within this hydrophobic patch attenuate the interaction with the Mlp1 C-terminal domain but do not alter the interaction with Gfd1, indicating that this patch forms a crucial component of the interface between Nab2 and Mlp1.

## Introduction

Gene expression depends on the production of mature mRNA transcripts that must be exported from their site of synthesis in the nucleus to the cytoplasm for translation. This complex multistep process can be conveniently subdivided into three distinct stages: generation of mature mRNP export complexes in the nucleus, their equilibration between the nucleus and the cytoplasm through nuclear pore complexes (NPCs), and disassembly of the export complex in the cytoplasm that prevents its return to the nucleus.^1–5^ The generation of export-competent mRNPs is a prerequisite for nuclear export and represents the culmination of transcription, addition of a 5′-cap to the transcript, splicing out of introns, and cleavage and polyadenylation of the 3′-end.^1,2^ Following completion of these processing steps, the mature transcript must be translocated to the cytoplasm where it can interface with the translation machinery. Recent evidence indicates that mRNA synthesis, processing, and export are coordinated.^2,6^ This coordination is achieved via a large number of mRNA-binding proteins that interact with the transcript from transcription through translation and ultimately to its destruction. The export of mature mRNPs through NPCs is mediated primarily by an evolutionarily conserved heterodimer, termed Mex67:Mtr2 in yeast, via its transient interactions with phenylalanine glycine repeat-containing proteins of the nuclear pore called FG nucleoporins.^1–3,5^ When the export complex reaches the cytoplasm, the RNA helicase, Dbp5, and Gle1 mediate its disassembly, and this process has been proposed to function as a molecular ratchet that prevents the return of the mRNP to the nucleus.^5,7–9^

Cells require an efficient mechanism to establish when mRNA is mature and competent for export. Thus, there are a number of cellular quality-control mechanisms that work actively to destroy improperly processed transcripts and preferentially export correctly processed mRNA to complement the cellular factors that assure coordinated production and export of correct mRNA transcripts.^2,10,11^ The nuclear exosome, a complex of 3′–5′ riboexonucleases, can detect and destroy aberrant or incorrectly processed transcripts within the nucleus.^12^ Several additional quality-control mechanisms function posttranscriptionally and include the nonsense-mediated decay pathway, which recognizes transcripts with defects such as premature stop codons,^13^ and the nonstop decay pathway, which recognizes transcripts lacking a stop codon.^14^ Recognition of transcripts by any of these pathways leads to their destruction. In addition to these “search-and-destroy” quality-control mechanisms in the nucleus and the cytoplasm, recent work has uncovered evidence in *Saccharomyces cerevisiae* that surveillance of mRNA can also occur at the nuclear pore.^2,10,15,16^ These studies suggest that specific interactions between mRNA-binding proteins associated with a given transcript and the evolutionarily conserved myosin-like protein 1 (Mlp1), located at the inner nuclear basket of the NPC,^17^ can influence nuclear export of that transcript. For example, if introns are retained in a transcript, interactions between splicing factors and Mlp1 lead to retention of the transcript in the nucleus.^16^

Heterogeneous nuclear ribonucleoproteins (hnRNPs) are important in orchestrating the different steps of the gene expression pathway.^1–3^ One important hnRNP function is to sort export-competent mRNPs and concentrate them in the vicinity of NPCs to facilitate export. *N*uclear abundant poly(*A*) RNA-*b*inding protein (Nab2) is an essential multidomain hnRNP that is required for the nuclear export of poly(A) mRNA in *S. cerevisiae*.^18,19^ Nab2 is also involved in poly(A) tail length control^19^ and has been proposed to provide a link between the termination of polyadenylation and mRNA nuclear export. Nab2 interacts with Mlp1, which is constructed from a long α-helical coiled coil and a globular C-terminal domain^20^ that binds to Nab2.^21^ Overexpression of Mlp1 or its C-terminal domain causes retention of both Nab2 and poly(A)^+^ RNA in the nucleus.^21^ Mlp1 is itself attached to the nucleoplasmic face of NPCs and, hence, is thought to facilitate coordination of several steps in mRNA metabolism,^15,16^ albeit the Mlp proteins are not essential for cell growth.^17^ Because it both recognizes the presence of a poly(A) tail and interacts with Mlp1, Nab2 is an attractive candidate to participate in the final step of the nuclear portion of the gene expression pathway.

**Fig. 1:**
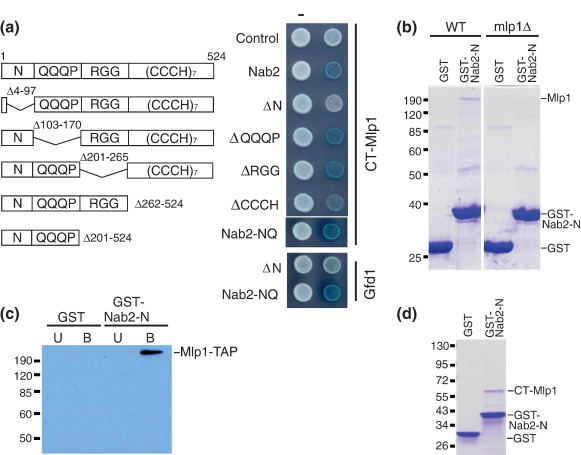
The N-terminal domain of Nab2 interacts with Mlp1. (a) A domain schematic of the Nab2 protein showing the Nab2 deletion mutants that were analyzed using the yeast two-hybrid assay. The relative locations of the N-terminal (N), QQQP repeat, RGG, and tandem zinc finger (CCCH) domains are indicated. The size (in amino acids) is indicated for the full-length protein, and the residues deleted from each variant are indicated. The yeast two-hybrid reporter strain (EGY48) was transformed with the indicated DBD plasmids expressing each Nab2 variant, in combination with an AD control plasmid (−), an AD plasmid expressing CT-Mlp1 (residues 1490–1875), or, as a control, an AD expressing Gfd1. Positive interactions are indicated by the blue color that arises from activation of the β-galactosidase (lacZ) reporter. (b) The N-terminal domain of Nab2 (Nab2-N) binds Mlp1 from yeast lysates. Purified recombinant GST-Nab2-N or GST alone as a control protein was incubated in yeast cell lysate prepared from either wild-type (WT) or *MLP1* deletion (*mlp1*Δ) cells. The GST proteins were purified on glutathione beads, and copurifying proteins were visualized by Coomassie Blue staining. A band corresponding to Mlp1 copurified with GST-N from wild-type but not *mlp1*Δ cells. (c) GST-Nab2-N or GST alone as a control protein was incubated in yeast lysate from cells expressing TAP-tagged Mlp1. GST fusion proteins were purified, and unbound (U) and bound (B) fractions were analyzed by SDS-PAGE and immunoblotting with PAP antibody to detect TAP-tagged Mlp1. (d) Purified recombinant GST-Nab2-N or GST control protein was incubated with Mlp1 C-terminal domain and binding was assessed by Coomassie Brilliant Blue staining.

Nab2 shuttles between the nucleus and the cytoplasm and is thought to interact with transcripts following 3′-end cleavage and addition of the poly(A) tail.^18,19,22^ Thus, Nab2 and other shuttling mRNA-binding proteins could serve as positive markers to target mature transcripts for preferential export from the nucleus. The Nab2 protein (Fig. 1a) has a modular structure based on four functional domains:^23^ a unique N-terminal domain of approximately 100 residues, a glutamine-rich linker, an RGG domain, and a Zn-finger domain. Deletion analysis indicates that the N-terminal domain is necessary for the nuclear export of both poly(A) RNA and Nab2 *in vivo*, consistent with a model in which Nab2 associates with mRNA in the nucleus and in which the N-terminal domain facilitates export of both poly(A) mRNA and Nab2.^23^ The RGG domain, which contains the nuclear localization sequence^24^ recognized by the nuclear import factor Kap104 (also known as transportin or Kapβ2), is required for the nuclear import of Nab2^25^ to recycle it for another round of mRNA export. The Zn-finger domain is critical for Nab2 binding to polyadenosine mRNA.^22,26^

The N-terminal domain of Nab2 interacts with the export factor, Gfd1,^27^ a protein that may be involved in the terminal step of the export pathway involving disassembly of the export complex in the cytoplasm by the RNA helicase, Dbp5, and the export factor, Gle1.^3,28^ Gfd1 is a high-copy suppressor of the *rat*8-2 allele of *DBP5* and binds to both Gle1 and the cytoplasmically oriented nuclear pore protein, Nup42,^28^ and, thus, has the potential to link Nab2 to both Gle1 and the cytoplasmic face of the NPC.^27^

Here, we show that the N-terminal domain of Nab2 interacts with the C-terminal domain of Mlp1. Our studies demonstrate that the N-terminal domain of Nab2 is both necessary and sufficient to mediate interaction with Mlp1. We have determined the atomic resolution structure of the Nab2 N-terminal domain, using both NMR and X-ray crystallography, and show that it is analogous to the PWI fold found in several RNA-binding proteins. We have identified a putative protein:protein interaction site containing a hydrophobic surface patch centered on Phe73 that mutagenesis indicates is involved in the interaction between Nab2 and Mlp1 but not in the interaction with Gfd1.

## Results and Discussion

### The Nab2 N-terminal domain binds to Mlp1

Previous work^21^ showed that full-length Nab2 binds to the C-terminal globular domain of Mlp1 (CT-Mlp1). We utilized the yeast two-hybrid system to identify the domain of Nab2 that interacts with CT-Mlp1. Nab2 can be divided into four domains,^23^ and hence, we constructed a series of two-hybrid bait plasmids expressing Nab2 in which each of the individual domains had been deleted (Fig. 1a). We used a lacZ reporter that generates a blue color when an interaction occurs.^29^ Each Nab2 plasmid was coexpressed with the vector alone (pJG4–5) or CT-Mlp1 (Fig. 1a). As shown in Fig. 1a, the negative vector controls (pEG202 and pJG4–5) were white, confirming that they did not activate the lacZ reporter. In contrast, coexpression of CT-Mlp1 and Nab2 generated the blue color, indicating that the lacZ reporter was activated, consistent with the proteins interacting with one another. CT-Mlp1 failed to interact with Nab2 only when the N-terminus was deleted (ΔN-Nab2), and the interaction was maintained with all other Nab2 fusion proteins. To determine if the N-terminal domain of Nab2 was sufficient for interaction with CT-Mlp1, we attempted to create a minimal yeast two-hybrid construct expressing only the N-terminal domain of Nab2. Unfortunately, this N-terminal domain fusion protein autoactivated the lacZ reporter; thus, we instead created a fusion consisting of both the N-terminal domain and the QQQP domain (Nab2-NQ). This protein interacted with CT-Mlp1 (Fig. 1a). Gfd1 was used as a positive control for interaction with the N-terminal domain of Nab2,^27^ and, as expected, Gfd1 interacted with the NQ domain but not with ΔN-Nab2 (Fig. 1a). For all two-hybrid experiments, expression of each fusion protein was confirmed by immunoblotting (data not shown). Overall, the two-hybrid data suggest strongly that the N-terminal domain of Nab2 is both necessary and sufficient to interact with the Mlp1 C-terminal domain.

Pull-down assays complemented the results obtained using the two-hybrid system and confirmed that the N-terminal domain of Nab2 without the QQQP domain is both necessary and sufficient to mediate interactions with Mlp1. A glutathione *S*-transferase (GST) fusion to the N-terminal domain of Nab2 (GST-Nab2-N, residues 1–97 of Nab2) was engineered, expressed in *Escherichia coli*, and purified. Either GST-Nab2-N or GST alone as a control protein was incubated in yeast lysate, and then, the GST protein and any associated proteins were bound to glutathione beads. As shown in Fig. 1b, a band of approximately 220 kDa that copurified with GST-Nab2-N but not with GST alone was visualized using Coomassie Blue staining. Immunoblotting confirmed that this band corresponded to the full-length Mlp1 protein (Fig. 1c). When the yeast lysate was prepared from *mlp1Δ* cells, this 220-kDa band was not detected by either Coomassie staining (Fig. 1b) or immunoblotting (Fig. 1c). Although the amount of Mlp1 copurified with GST-Nab2-N from yeast lysate was rather small, it is quite remarkable that a band corresponding to this full-length, very large, nuclear pore-associated protein^17^ can be isolated from yeast extract in a single step. The results of this experiment indicate that the N-terminal domain of Nab2 is sufficient to copurify Mlp1 from a complex mixture, showing that the interaction is highly specific.

An *in vitro* binding assay established that the N-terminal domain of Nab2 interacted directly with the Mlp1 C-terminus. Recombinant proteins were expressed and purified from *E. coli* as described in Materials and Methods. Either GST-Nab2-N or GST control protein was incubated with the Mlp1 C-terminal domain fragment. As shown in Fig. 1d, the Mlp1 fragment bound to GST-Nab2-N but not to the control GST protein, confirming that the N-terminal domain of Nab2 interacts directly with the C-terminal domain of Mlp1. The amount of binding between Nab2-N and CT-Mlp1 appears to be substoichiometric, which is consistent with the interaction between Nab2 and Mlp1 being relatively weak, to facilitate export rather than retention at the nuclear pore. Preliminary binding studies indicated that the *K*_d_ for this interaction is in the micromolar range (data not shown).

### Structure of the Nab2 N-terminal domain

**Fig. 2:**
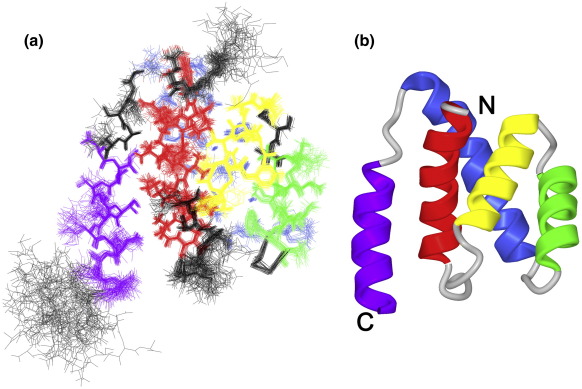
Solution structure of the Nab2 N-terminal domain. (a) Ensemble view of the 45 accepted structures, showing all heavy atoms (superposed using the N, C^α^, and C′ atoms of residues 4–99). The backbone and the majority of side chains are determined to high precision, with significant disorder being limited to the chain termini, a few surface side chains, and the loops between helices 1 and 2 and between helices 4 and 5. Structural statistics appear in Table 1. (b) A schematic illustration of the secondary structure of the N-terminal domain of Nab2. The fold of this domain is based on five α-helices (H1, red; H2, yellow; H3, green; H4, blue; and H5, purple) that pack to form a compact bundle. The arrangement of helices H1–H4 is analogous to that of the PWI fold^30^ (see Fig. 4).

The structure of the N-terminal domain of Nab2 was established using both NMR and X-ray crystallography. A fragment corresponding to residues 1–105 of Nab2 was produced in large quantities by bacterial expression and purified using conventional ion-exchange and gel-filtration chromatographic methods (see Materials and Methods). This material was exceptionally soluble, which facilitated the collection of NMR spectra, from which a model was generated using conventional methods. The final ensemble of 45 calculated structures of the Nab2 N-terminal domain is shown in Fig. 2a. Of the total of 50 structures calculated in the final round, these 45 correspond to a well-defined plateau region in the energy and energy-ordered root-mean-square deviation (rmsd) profiles (Fig. 2a), indicating that they form a suitable set for reporting structural statistics (Table 1).^31^ Residues Met1–Gln3 and Gly100–Ala105 were unstructured in solution, as evidenced by the lack of any medium- or long-range nuclear Overhauser enhancement (NOE) connectivities for these residues and by the sharpness and near random-coil chemical shift values of their NMR resonances. The remainder of the domain was well ordered, although there was slightly increased disorder in the loops, especially those between helix 1 and helix 2 (Pro22–Asp27) and, to a lesser extent, between helix 3 and helix 4 (Asp57–Ser60). The backbone rmsd over residues 4–99 was 0.47 ± 0.13 Å.

As illustrated in Fig. 2b, the solution structure of the N-terminal domain of Nab2 was based on a bundle of five α-helices (H1–H5). Unlike the highly compact bundle formed by helices H1–H4, each of which made multiple contacts with at least two of the other helices, helix H5 was less tightly associated with the rest of the structure. The only NOE constraints from helix H5 to other parts of the structure were all contacts to helix H1 (Ala84 contacts Asn9, Val12, and Ile13; Ile87 contacts Ile13 and Glu16; Ile91 contacts Glu16 and Ala19; Asn95 contacts Ala19 and Gly20). These contacts were sufficient to constrain the position of helix H5 with comparable precision to those of the other helices, but they were much fewer in number than the constraints involving any of the other helices. Further evidence for the relatively loose attachment of helix H5 to the rest of the structure came from the observation that NMR samples were slowly proteolyzed from the C-terminus. Over a period of several weeks, many signals assigned to residues of helix H5 were progressively lost from the spectra, whereas the signals assigned to helices H1–H4 were largely unaffected. The signals for protons in helix H5 were generally weaker than other components of the structure and decreased with time, consistent with its being gradually proteolyzed.

**Fig. 3:**
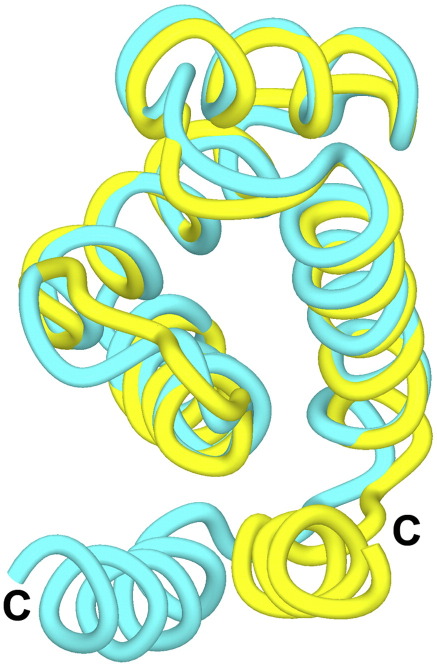
Crystal structure of the Nab2 N-terminal domain and comparison with the solution structure. Helices H1–H4 of the solution (cyan) and crystal (yellow) structures of the N-terminal domain of Nab2 superimposed well, but the position of helix H5 changed substantially between the two. In the solution structure, this helix appeared to be less intimately associated with the rest of the domain. The change in the position of helix H5 may be due to the removal of residues 95–99 from this helix in the crystal structure or might be the result of crystal packing.

We also obtained *P*4_3_2_1_2 symmetry crystals of the Nab2 N-terminal domain that diffracted past 1.8 Å resolution in-house (Table 2). We used the solution NMR structure of this domain as a model for molecular replacement and, after trying a number of different models containing different fragments of the structure, were able to obtain a unique solution using residues 7–82 (corresponding to helices H1–H4). After solvent flattening, the electron density enabled residues 6–94 to be built to produce a structural model that had an *R*-factor of 20.7% (*R*_free_ = 26.4%) and excellent geometry (Table 2) after iterative cycles of refinement and rebuilding (Fig. 3).

### Comparison between solution and crystal structures

**Fig. 4:**
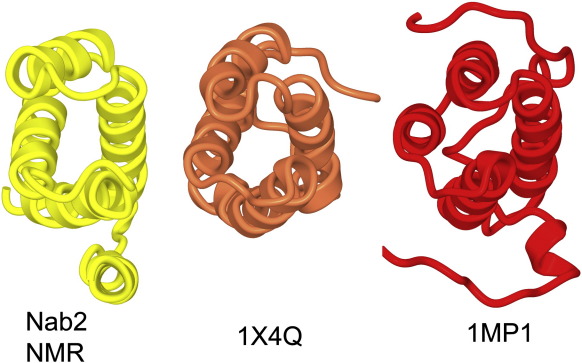
Comparison of the structure of the solution structure of the Nab2 N-terminal domain with PWI folds seen in the solution structures of SRm160 (PDB accession code 1MP1) and the U4/U6 small nuclear ribonucleoprotein, Prp3 (PDB accession code 1X4Q). The arrangement of helices H1–H4 of the Nab2 N-terminal domain is similar to that of the PWI folds, although these do not contain an element analogous to helix H5.

The crystal and solution structures of the N-terminal domain of Nab2 were very similar for helices H1–H4 and superimposed with a C^α^ rmsd of 1.2 Å. However, the position of helix H5 varied significantly between the two structures as a result of a rigid-body rotation of the order of 25° (Fig. 4). This difference in the position of helix H5 could have been the result of crystal packing or might have been a consequence of partial proteolysis as it was only possible to trace the chain in the crystal structure as far as residue 94, whereas in the solution structure, this helix extended to residue 99. However, these differences are also consistent with the helix being only loosely associated with the rest of the structure, which is also consistent with the relatively few NOE connectivities to helices H1–H4 in the NMR data. Moreover, when the TLS (translation/libration/screw) rigid-body motion^32^ of the Nab2 N-terminal domain was modeled by assigning helices H1–H4 to one rigid body and helix H5 to another, the *R*-factor and *R*_free_ derived from X-ray crystallography dropped substantially to 19.5% (from 20.7%) and 25.0% (from 26.4%), respectively. Analysis of the TLS motion using the TLDMD method^33^ indicated that the libration motions of helix H5 (20.1°^2^) were substantially greater than those of the helix H1–H4 core (libration of 4.6°^2^), consistent with helix H5 being substantially more mobile. Potentially, the movement of helix H5 might have a regulatory role in modulating the interactions between the Nab2 N-terminal domain and other components of the mRNA export machinery.

### Similarity to the PWI fold

**Fig. 5:**
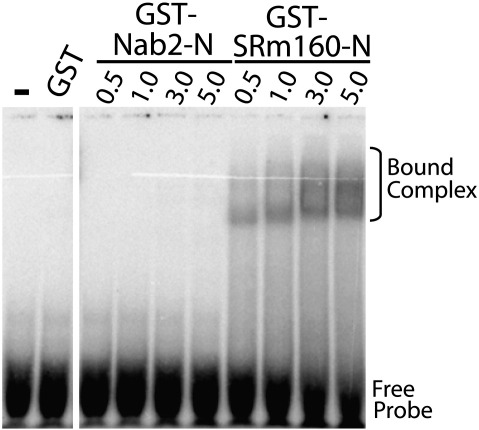
The N-terminal domain of Nab2 does not bind to RNA. Binding to radioactively labeled randomized sequence 25-nt poly(N) RNA probe was assessed by a gel shift assay. Purified recombinant GST-Nab2-N, negative control, GST alone, or positive control, GST-SRm160-N, was incubated with radioactively labeled RNA probe. Complexes were resolved by native gel electrophoresis. Results are shown for probe alone (−), GST alone, or increasing concentrations (0.5–5 μM) of GST-Nab2-N or GST-SRm160-N. The positions of the free probe and bound complexes are indicated.

A search for structural homologues of the Nab2 N-terminal domain using the DALI web site† indicated that it had distant similarity to the PWI domain fold (Fig. 4) that is found in a number of nucleic acid binding proteins such as the splicing factor and exon junction component SRm160.^30,34^ The Pro-Trp-Ile sequence for which the PWI domain is named is found in helix H1 of this fold and is important in generating the hydrophobic core of the PWI fold in which the Trp and Ile side chains pack against Phe101 in helix H4 of SRm160. The PWI fold is thought to bind both single- and double-stranded DNA and RNA through a characteristic basic patch on its surface.^34^ However, there is little conservation at the sequence level, and the characteristic Pro-Trp-Ile (PWI) sequence motif is not present in the Nab2 N-terminal domain where the corresponding residues are Val12, Ile13, and Val14. Similarly, Nab2 lacks the characteristic positively charged surface patch that has been associated with nucleic acid binding.^34^ Moreover, the nucleic acid binding function of SRm160 also requires residues upstream of the PWI domain^34^ that are lacking in the Nab2 N-terminal domain, and, consistent with this observation, we were unable to detect any interaction between the Nab2 N-terminal domain and poly(N) RNA in band-shift assays under conditions where the PWI domain from SRm160 showed a clear interaction (Fig. 5).

### Putative protein binding site centered on Phe73

**Fig. 6:**
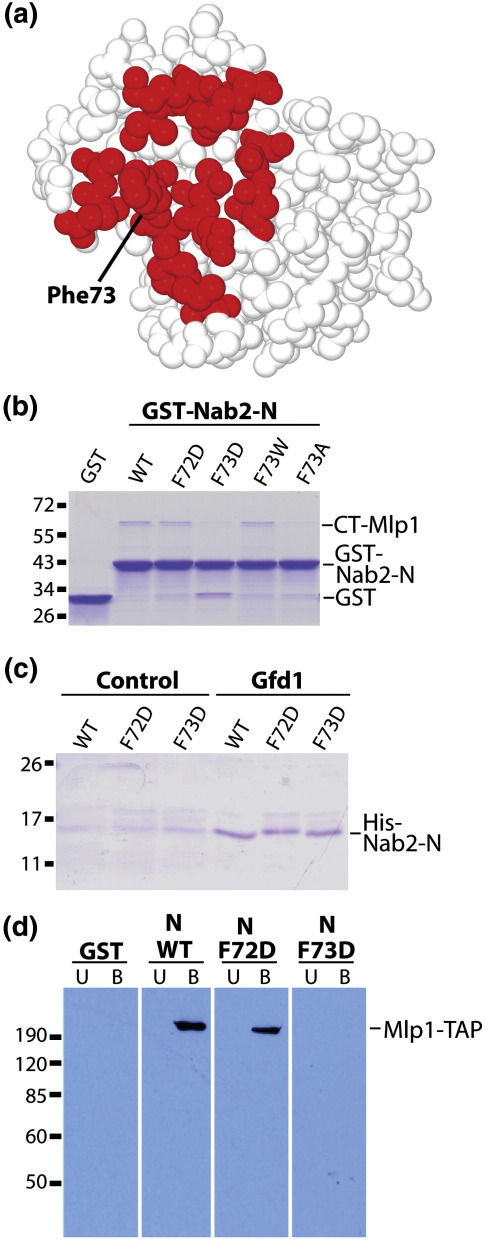
A hydrophobic pocket on Nab2 is required for the interaction with Mlp1 but not with Gfd1. (a) Illustration of the hydrophobic putative protein interaction site (red) on the surface of the Nab2 N-terminal domain centered on Phe73 identified by the PPI-Pred site [http://www.bioinformatics.leeds.ac.uk/ppi-pred/]. The aromatic ring of Phe73 projects away from the protein surface and, hence, is an attractive candidate for a component of an interaction surface. (b) Wild-type (WT) or mutant (F72D, F73D, F73W, and F73A) GST-Nab2-N proteins were incubated with purified recombinant Mlp1 C-terminal domain. GST alone serves as a specificity control. GST fusion proteins were purified on glutathione beads and then eluted with sample buffer. Bound proteins were visualized by Coomassie Blue staining. (c) Wild-type (WT) or mutant (F72D and F73D) purified recombinant His-tagged Nab2-N was incubated with either Gfd1 beads (right) or control ovalbumin beads. Proteins bound to the beads were eluted with sample buffer, and the bound proteins were resolved by PAGE and visualized by Coomassie Blue staining. Note that Gfd1 and the control protein, ovalbumin, are covalently attached to beads; hence, they are not present in the bound fraction eluted with sample buffer. (d) Wild-type or mutant (F72D and F73D) GST-Nab2-N was incubated in yeast lysate prepared from cells expressing Mlp1-TAP. As a control, GST alone was also analyzed. Proteins that copurified with the GST fusion proteins on glutathione beads were analyzed by immunoblotting with PAP antibody, which detects the TAP-tagged Mlp1 protein.

Inspection of the solution and crystal structures of the Nab2 N-terminal domain showed the presence of a hydrophobic patch centered on Phe72 and Phe73 (Fig. 6a), suggesting that this region of the surface might represent a putative protein:protein interaction interface. Phe73 in particular was completely exposed on the surface of the protein. We therefore engineered a series of amino acid changes in these positions and assayed these mutants for binding to Mlp1 and Gfd1. Mutation of Phe72 had a negligible effect on the binding of the Nab2 N-terminal domain to either the Mlp1 C-terminal fragment or the Gfd1 C-terminus (Fig. 6b–d). In contrast, mutation of Phe73 to Asp or Ala dramatically reduced the affinity of Nab2 for the Mlp1 C-terminal fragment, whereas mutation to Trp did not decrease its affinity (Fig. 6b). Significantly, these mutations did not alter the binding of the Nab2 N-terminal domain to the Gfd1 C-terminal fragment, consistent with the mutations not altering the overall conformation of the domain (Fig. 6c). Binding experiments using yeast lysates confirmed that mutations in the hydrophobic patch on Nab2 interfered with the interaction between Nab2-N and the intact full-length Mlp1 protein. Equal amounts of GST-Nab2-N variants (WT, F72D, and F73D) were incubated in yeast lysate prepared from cells expressing protein-A-tagged Mlp1, and then, proteins associated with GST-Nab2-N were recovered using glutathione beads. Mlp1 that copurified with GST-Nab2-N was detected by immunoblotting for the protein A tag. As shown in Fig. 6d, full-length Mlp1 copurified with both wild-type Nab2-N and F72D Nab2-N. However, copurification of Mlp1 was not detected with either the GST control or the Nab2-N F73D mutant. Overall, the mutagenesis results are consistent with Phe73 forming a crucial component of the interaction interface between Nab2 and Mlp1 and hydrophobic interactions making an important contribution to the interface.

In summary, our results show that the N-terminal domain of Nab2 binds to the C-terminal domain of Mlp1 and that the Mlp1 binding site on Nab2 does not appear to overlap with the Gfd1 binding site. The Nab2 N-terminal domain has a fold based on a five-helix bundle that is analogous to the PWI fold found in SRm160 and other nucleic acid binding proteins, although it does not retain the nucleic acid binding function seen in other PWI domains. The hydrophobic side chain of Phe73 in the Nab2 N-terminal domain is exposed on the surface of the molecule and appears to be a crucial component of the Mlp1 binding site, although, clearly, further work will be required to define the precise nature of the interface between these two molecules. Our results are consistent with a model that envisages that interaction with Mlp proteins occurs soon before mRNA is exported and that Nab2 is important either for targeting the mRNA to the Mlp proteins or for releasing the mature mRNA from the Mlp proteins to enable its transit through the NPC. Such a model would be consistent with the increased nuclear retention of mRNA seen on overexpression of Mlp1 *in vivo*.^21^

## Materials and Methods

### Strains, plasmids, and chemicals

All DNA manipulations were performed according to standard methods,^35^ and all media were prepared by standard procedures.^36^ All yeast strains and plasmids used are described in Table 3. Chemicals were obtained from Ambion (Austin, TX), Sigma Chemical Co. (St. Louis, MO), US Biological (Swampscott, MA), or Fisher Scientific (Pittsburgh, PA) unless otherwise noted. Site-directed mutagenesis was performed using the Stratagene QuikChange mutagenesis kit. All mutants were sequenced to ensure that the intended change was introduced and no additional changes to the sequence were present.

### Yeast two-hybrid analysis

The yeast two-hybrid reporter strain (EGY48) containing the DNA-binding domain (DBD) vector (pEG202)^29^ or the appropriate DBD fusion protein plasmids was transformed with the activation domain (AD) vector (pJG4–5)^29^ or the indicated AD fusion protein plasmids. Briefly, transformants were tested for a positive yeast two-hybrid interaction by expression of lacZ and galactose-dependent growth on media lacking leucine. Cells were grown at 30 °C on glucose or galactose minimal media, minimal media lacking leucine, or minimal media containing 200 μM 5-bromo-4-chloro-3-indolyl-β-d-galactosidase. The expression of the appropriately sized fusion proteins was confirmed by immunoblotting using either LexA antibody (1:1000 dilution, Covance) or HA antibody (1:1000 dilution, Santa Cruz).

### *In vitro* binding assays

For *in vitro* assays, purified recombinant Nab2-N (amino acids 1–97) and CT-Mlp1 (amino acids 1490–1779) were employed. Nab2-N was expressed as a tobacco etch virus protease-cleavable GST fusion protein to assess direct binding.^37^ GST-Nab2-N (pAC2058) was expressed in *E. coli* DE3 cells. Cells were collected and lysed in phosphate-buffered saline (PBS) (137 mM NaCl, 10 mM phosphate, and 2.7 mM KCl, pH 7.4) supplemented with protease inhibitor mixture (1 mM PMSF, 3 ng/ml pepstatin A, leupeptin, aprotinin, and chymostatin) by incubation with lysozyme (0.1 mg/ml) for 30 min on ice followed by sonication to purify the GST fusion proteins. Lysates were clarified by centrifugation and incubated with glutathione Sepharose (Amersham) in buffer A [20 mM Tris–HCl, pH 8.0, 100 mM NaCl, 1 mM ethylenediaminetetraacetic acid (EDTA), 1 mM β-mercaptoethanol, 1 mM PMSF, and 0.1% Igepal] for 2 h at 4 °C with mixing. The beads were then washed with PBS and 0.5% Triton X-100.

His-CT-Mlp1 (pAC1486) was expressed in *E. coli* DE3 cells. Cells were collected and lysed in lysis buffer (50 mM NaH_2_PO_4_, 300 mM NaCl, and 10 mM imidazole, pH 7.0) supplemented with protease inhibitor mixture by incubation with lysozyme and sonication. Lysates were clarified by centrifugation and incubated with Ni–NTA agarose (Qiagen) in lysis buffer for 2 h at 4 °C with mixing. The beads were then washed with wash buffer (50 mM NaH_2_PO_4_, 300 mM NaCl, and 20 mM imidazole). His-Nab2 and His-CT-Mlp1 were eluted from agarose with 250 mM imidazole. Sepharose-bound GST or GST-CT-Mlp1 (6 μg) was incubated with 2 μg of purified His-Nab2 at 4 °C in PBS for 90 min. Sepharose-bound GST-NT-Nab2 WT, F72D, or F73D (6 μg) was incubated with purified His-CT-Mlp1 (2 μg). Unbound fractions were collected and the beads were washed three times with PBS. Bound fractions were eluted with sample buffer (50 mM Tris–HCl, pH 6.8, 2% SDS, 10% glycerol, 1% β-mercaptoethanol, 12.5 mM EDTA, and 0.02% bromophenol blue) and analyzed by SDS-PAGE followed by Coomassie Blue staining.

Each GST-Nab2-N fusion protein or the GST control protein was added to yeast lysate to examine the interaction between the N-terminal domain of Nab2 and full-length Mlp1. GST fusion proteins were then purified on glutathione beads, and copurifying proteins were visualized either by Coomassie Blue staining or by immunoblotting.

Purified recombinant His-tagged Gfd1 was prepared as previously described to examine the interaction between Nab2-N and Gfd1.^27^ The Gfd1 protein was attached to CNBr Sepharose beads as previously described.^38^ Briefly, CNBr Sepharose beads (Amersham Pharmacia Biotech) were swollen and washed in 1 mM HCl. Beads were transferred to coupling buffer (100 mM NaHCO_3_, pH 8.3, and 500 mM NaCl) and added to 2–5 mg of Gfd1 in coupling buffer. Coupling was carried out at 4 °C overnight. Residual active groups were blocked with 1 M Tris–HCl, pH 8.0, for 2 h at room temperature. Beads were then washed successively and extensively four times in coupling buffer and acid wash buffer (0.1 M sodium acetate, pH 4.0, and 500 mM NaCl). For binding assays, 10 μg of Nab2-N was incubated with 50 μl of Gfd1 beads for 2 h at 4 °C. Beads were then washed twice in PBS, and bound proteins were eluted with 100 μl of sample buffer. Samples were resolved by PAGE, and bound proteins were detected by Coomassie Blue staining.

### Expression and purification of the N-terminal domain of Nab2 for structural studies

Residues 1–105 of Nab2 were cloned into pET28a and pET30a vectors (Novagen) via NdeI and BamHI sites to produce a His-tagged and untagged construct, respectively. Proteins were expressed in *E. coli* BL21 (DE3) CodonPlus RIL cells by overnight induction with 1 mM IPTG at 25 °C. His-tagged Nab2 1–105 was purified by using Ni–NTA agarose resin according to manufacturer's instructions (Qiagen Inc.) followed by gel filtration on a Superdex 75 HiLoad 26/60 prep grade column (GE Healthcare) with 20 mM Tris–HCl, pH 8.0, 1 mM DTT, 1 mM EDTA, and 50 mM NaCl as buffer. Untagged protein was purified by anion-exchange chromatography (Q-Sepharose HiLoad 16/10 column, GE Healthcare) applying in 20 mM Bis-Tris–HCl, pH 6.0, 1 mM DTT, and 1 mM EDTA and eluting with a linear gradient of NaCl from 0 to 500 mM in the same buffer followed by gel filtration chromatography as above. Typical yields were 40 mg/l of culture. The resultant proteins were > 95% pure as assessed by SDS-PAGE. By mass spectrometry, the untagged material had an *M*_r_ of 11,324.6, consistent with Met1 having been removed (theoretical *M*_r_ = 11,455.2 for full length, with *M*_r_ = 11,324.0 corresponding to loss of the N-terminal Met), whereas the His-tagged construct had an *M*_r_ of 13,489 (theoretical *M*_r_ = 13487.2, corresponding to loss of the N-terminal Met). Nab2 dual labeled with ^15^N and ^13^C was purified from cultures harboring pET30a:Nab2 (1–105) grown in M9 minimal medium supplemented with ^13^C-labeled glucose and ^15^N-labeled NH_4_Cl.

### Solution structure

NMR spectra were acquired at 27 °C on Bruker Avance 800 and DRX500 spectrometers, equipped with triple-resonance (^1^H/^15^N/^13^C) cryoprobes. All data were acquired using a sample containing 2 mM ^15^N/^13^C-labeled protein, 25 mM sodium phosphate, pH 6.0, 10 mM sodium chloride, and 10% ^2^H_2_O and comprised the following: 2D: [^15^N–^1^H] heteronuclear single quantum coherence (HSQC), [^13^C–^1^H] HSQC covering full ^13^C spectral width, constant-time [^13^C–^1^H] HSQC covering only aliphatic ^13^C region, [^1^H–^1^H] NOE spectroscopy (NOESY) experiments (τ_m_ = 50, 100, and 150 ms), [^1^H–^1^H] NOESY experiments filtered to remove ^15^N-coupled signals in F_2_ (τ_m_ = 50 and 150 ms); 3D data sets: CBCANH, CBCACONH, HBHANH, HBHACONH, [^1^H–^13^C–^1^H] HCCH–total correlated spectroscopy, [^1^H–^13^C–^1^H] HCCH–correlated spectroscopy, ^15^N NOESY–HSQC (τ_m_ = 50 and 150 ms), and ^13^C NOESY–HSQC (τ_m_ = 50 and 150 ms), with separate data sets acquired for ^13^C aliphatic and aromatic spectral regions. ^1^H, ^15^N, and ^13^C chemical shifts were calibrated using sodium 3,3,3-trimethylsilylpropionate as external ^1^H reference.^39^

NOE distance restraints were derived from analysis of all of the data from NOE-based experiments. Cross-peak intensities were measured using the program SPARKY^40^ and grouped into four categories. The strongest dα_N_ (*i*, *i* + 1) were used to set the upper limit for the category “very strong” (0–2.3 Å), strong d_NN_ (*i*, *i* + 1) connectivities in α-helices defined the category “strong” (0–2.8 Å), dα_N_ (*i*, *i* + 3) cross peaks in helices defined the category “medium” (0–3.5 Å), and all remaining peaks were classified as “weak” (0–5 Å). Lower bounds for all NOE restraints were set to zero,^41^ and no multiplicity corrections were required since *r*^− 6^ summation was used for restraints involving groups of equivalent or nonstereoassigned spins.^33,42^

Structures were calculated from polypeptide chains with randomized ϕ and ψ torsion angles using a two-stage simulated annealing protocol within the program XPLOR, essentially as described elsewhere,^43,44^ but employing larger numbers of cycles as follows: first-stage calculations comprised Powell energy minimization (500 steps), dynamics at 1000 K (25,000 steps), increase of the van der Waals force constant and tilting of the NOE potential function asymptote (4000 steps), switching to a square-well NOE function then cooling to 300 K in 2000 step cycles, and final Powell minimization (1000 steps). Second-stage calculations used Powell minimization (500 steps), increasing dihedral force constant during 4000 step cycles of dynamics at 1000 K (with a strong van der Waals force constant and square-well NOE potential function), cooling to 300 K in 1000 step cycles, and 2000 steps of final Powell minimization.

The program CLUSTERPOSE was used to calculate the mean rmsd of ensembles to their mean structure.^45,46^

### Crystallography

Crystals were obtained by vapor diffusion after 18 months from a 60-mg/ml solution of His-tagged Nab2 (residues 1–105) with a well buffer containing 0.2 M MgCl_2_, 0.1 M Tris–HCl, pH 8.5, and 20% (w/v) polyethylene glycol 4000. Crystals were cryoprotected in well buffer containing 20% glycerol and vitrified in a stream of anhydrous nitrogen at 100 K. A 1.8-Å-resolution data set (Table 3) was collected at 100 K in-house using a Rigaku X-ray generator equipped with Osmic mirrors and a MARdtb image plate detector. Data were processed using MOSFLM^45^ and reduced using SCALA and TRUNCATE.^48^ A molecular replacement was obtained using Phaser^48^ using the average NMR structure for residues 6–82 as a structural model. After solvent flattening using DM,^48^ the best molecular replacement solution was used as a starting model in ArpWarp^48^ that successfully built residues 6–94. After iterative cycles of refinement with REFMAC5 and model building using O^49^ and the addition of 59 water molecules, a final structure with an *R*-factor of 20.7% (*R*_free_ = 26.4%) and excellent geometry was produced (Table 2). Modeling TLS rigid-body motion based on helices H1–H4 and helix H5 reduced the *R*-factor to 19.2% (*R*_free_ = 25.0%).

### Gel shift RNA binding assay

Synthetic 25-nt poly(N) RNAs (Dharmacon) were 5′-end labeled with [γ-^32^P]ATP (GE Healthcare Life Sciences) using T4 polynucleotide kinase (Promega). The reaction was stopped, and unincorporated nucleotides were removed using Qiagen's Nucleotide Removal Kit. GST, GST-Nab2-N, and, as a positive control, GST-Srm160-N^35^ were expressed in *E. coli* and purified using glutathione beads as described above for *in vitro* binding assays. RNA electrophoretic mobility shift assays were performed by incubating the increasing amounts (0.5–5 μM) of recombinant GST, GST-Nab2-N, or GST-Srm160-N with approximately 30 pM radioactively labeled poly(N) RNA oligonucleotide (a 25-mer RNA oligonucleotide with each position randomized) in binding buffer for 30 min at 20 °C. Binding reactions were loaded onto a 5% native polyacrylamide gel and electrophoresed at 30 mA in 0.3× TBE buffer for 30 min to separate free oligonucleotide from protein–RNA complexes. Gels were dried and exposed overnight using a phosphorimager (Amersham).

### Data deposition

^1^H, ^13^C, and ^15^N NMR resonance assignments for the Nab2 N-terminal domain have been deposited at the BioMagResBank under accession code 15263, and the coordinates of the 45 accepted structures have been deposited under the Protein Data Bank (PDB) accession code 2JPS. Coordinates and structure factors for the crystal structure of the Nab2 N-terminal domain have been deposited with the PDB under accession codes 2V75 and R2V75SF, respectively.

## Figures and Tables

**Table 1:** Statistical data relating to the final ensemble of structures of the Nab2 N-terminus

Structural restraints		
*NOE-derived distance restraints*		
Intraresidue	439	
Sequential	325	
Medium (2 ≤ ∣*i* − *j*∣ ≤ 4)	337	
Long (∣*i* − *j*∣> 4)	140	
Total	1241	

*Cross-peak intensity categories*		
Very strong (0–2.3 Å)	23	
Strong (0–2.8 Å)	219	
Medium (0–3.5 Å)	641	
Weak (0–5.0 Å)	358	
Total	1241	

Statistics for accepted structures		
Number of accepted structures	45	
CNS energy terms (kcal mol^− 1^) (mean ± SD)		
*E* (total)	633.1 ± 11.3	
*E* (van der Waals)	307.5 ± 3.9	
*E* (distance restraints)	35.6 ± 3.9	
No distance violations greater than 0.35 Å		
rmsd from the ideal geometry used within XPLOR		
Bond lengths (Å)	0.0065	
Bond angles (°)	0.68	
Improper angles (°)	0.37	

Ramachandran statistics (%)		
	Residues 4–84	Residues 4–99

Most favored	93.5	94.0
Additionally allowed	6.1	5.6
Generously allowed	0.2	0.2
Disallowed	0.2	0.1

Atomic rmsd (Å) from the average structure (mean ± SD)		
	Residues 4–84	Residues 4–99

N, C^α^, C atoms	0.41 ± 0.13	0.47 ± 0.13
All heavy atoms	0.92 ± 0.15	0.97 ± 0.13

**Table 2:** Crystallographic statistics

*Data collection*	
Space group	*P*4_3_2_1_2
Unit cell dimensions (Å)	*a* = 48.14, *b* = 48.14, *c* = 81.20
Resolution range (Å)^a^	20–1.8 (1.9–1.8)
Total observations^a^	29,839 (3685)
Unique reflections^a^	9015 (1252)
Completeness (%)^a^	96.4 (94.2)
Multiplicity^a^	3.3 (2.9)
*R*_merge_ (%)^a^	4.1 (39.4)
*I*/σ(*I*)^a^	19.1 (3.3)

*Refinement*	
Number of reflections (working, test)	8556 (424)
*R*_cryst_/*R*_free_ (%)	19.2/25.0
Total number of non-H atoms	744
Number of water molecules	59
rmsd from ideal bond length (Å)	0.016
rmsd from ideal bond angles (°)	1.7
Ramachandran plot (%)	
Core region	96.3
Allowed region	3.7
Generously allowed region	0
Disallowed region	0

a Parentheses refer to final resolution shell.

**Table 3:** Yeast strains and plasmids used for this study

Strain/Plasmid	Description	Source/Reference
*Yeast strains*		
W303(ACY233)	*MATαura3*Δ *leu2*Δ *trp1*Δ *his3*Δ	Winston *et al.*^47^
*MLP1-TAP* (ACY984)	*MATa his3*Δ *leu2*Δ *met15*Δ *ura3*Δ *MLP1-TAP::HIS3*	Open Biosystems
*MLP1Δ* (ACY481)	*MATa leu2*Δ *ura3*Δ *his3*Δ *MLP1::HIS3*	Euroscarf

*Plasmids*		
pGEX4T-1	*GST, AMP*^*R*^*, bacterial expression vector*	Invitrogen
pAC2058	*GST-Nab2-N(aa 1–97) in pGEX4T-1, AMP*^*R*^	This study
pAC2349	*GST-Nab2-N F72D(aa 1–97) in pGEX4T-1, AMP*^*R*^	This study
pAC2350	*GST-Nab2-N F73D(aa 1–97) in pGEX4T-1, AMP*^*R*^	This study

## Footnotes

† http://www.ebi.ac.uk/dali/
